# Validation List no. 222. Valid publication of new names and new combinations effectively published outside the IJSEM

**DOI:** 10.1099/ijsem.0.006640

**Published:** 2025-03-31

**Authors:** Aharon Oren, Markus Göker

**Affiliations:** 1 The Institute of Life Sciences, The Hebrew University of Jerusalem, The Edmond J. Safra Campus, 9190401 Jerusalem, Israel; 2 Leibniz Institute DSMZ – German Collection of Microorganisms and Cell Cultures, Inhoffenstrasse 7B, 38124 Braunschweig, Germany

The purpose of this list is to publish in the *International Journal of Systematic and Evolutionary Microbiology (IJSEM)* those new names and new combinations that were effectively published outside the *IJSEM* as set forth in Rule 27 of the International Code of Nomenclature of Prokaryotes (ICNP; 2022 Revision) [1]. Authors and other individuals wishing to have new names and/or combinations included in future lists should send an electronic copy of the published paper to the *IJSEM* Editorial Office for confirmation that all of the other requirements for valid publication have been met. It is also a requirement of the *IJSEM* and ICNP that authors of new species, new subspecies and new combinations provide evidence that types are deposited in two publicly accessible culture collections in two different countries. It should be noted that the date of valid publication of these new names and combinations is the date of publication of this list, rather than the date of the original publication of the names and combinations. The authors of the new names and combinations are given below.

The inclusion of a name on these lists validates the publication of the name and thereby makes it available in the nomenclature of prokaryotes. The inclusion of a name on these lists is part of the requirements of valid publication of an effectively published name. Indeed, some of these names may, in time, be considered later synonyms, or it may be proposed to transfer the organisms to another genus, thus necessitating the creation of a new combination. Conversely, it may be proposed to transfer an organism back to an earlier genus and to regard the basonym or an earlier new combination as the correct name instead of a more recent new combination. The ICNP does not decide on such taxonomic opinions.

**Table:** 

Name/authors	Proposed as	Nomenclatural type^1^	Priority^2^	Reference
*Acidisoma cladoniae* Cho *et al*. 2025, 8^3,4^	sp. nov.	PAMC 29798 (=JCM 35634=KCTC 82159)	30	[2]
*Acinetobacter corruptisaponis* Wang *et al*. 2024, 8^3^	sp. nov.	DM2021935 (=GDMCC 1.3703=KCTC 92772)	3	[3]
*Acinetobacter thermotolerans* Shestivska *et al*. 2024, 9^3^	sp. nov.	ANC 7454 (=CCM 9356=CCUG 77195=CNCTC 8200)	34	[4]
*Adlercreutzia murintestinalis* Afrizal *et al*. 2022, e13^3^	sp. nov.	CLA-AA-M17 (=DSM 112345=JCM 37185)	29	[5]
*Alicyclobacillus acidocaldarius* subsp. *acidocaldarius* (Darland and Brock 1971) Nicolaus *et al*. 2002	subsp. nov. [basonym: *Bacillus acidocaldarius* Darland and Brock 1971 (Approved Lists 1980)]	104-1A (=ATCC 27009=BCRC 14685=CCRC 14685=CCUG 28521=CIP 106131=DSM 446=IFO 15652=JCM 5260=KCTC 1825=NBRC 15652=NCCB 89167=NCIMB 11725=NRRL B-14509)	15	[6]
*Alistipes muris* Afrizal *et al*. 2022, e13^3^	sp. nov.	CLA-AA-M12 (=DSM 112343=JCM 37184)	29	[5]
*Alkalispirillum* Rijkenberg *et al*. 2001, 374^5^	gen. nov.	*Alkalispirillum mobile* ^6^	27	[7]
*Alkalispirillum mobile* Rijkenberg *et al*. 2001, 374	sp. nov.	DSM12769 (=DSM 12769=VKM B-3569)	27	[7]
*Aquimarina litoralis* Oh *et al*. 2010, 435^7^	sp. nov.	CNURIC011 (=JCM 15974=KCTC 22614)	20	[8]
*Caldinitratiruptor* Fardeau *et al*. 2010, 246	gen. nov.	*Caldinitratiruptor microaerophilus*	21	[9]
*Caldinitratiruptor microaerophilus* Fardeau *et al*. 2010, 246	sp. nov.	CA62N (=DSM 22660=JCM 16183)	21	[9]
*Carboxydichorda* corrig. Karnachuk *et al*. 2024, 10^3,8^	gen. nov.	*Carboxydichorda subterranea*	33	[10]
*Carboxydichorda subterranea* corrig. Karnachuk *et al*. 2024, 10^3^	sp. nov.	L945 (=JCM 39509=VKM B-3704)	33	[10]
*Cellulosimicrobium arenosum* Oh *et al*. 2018, 903^9^	sp. nov.	CAU 1455 (=KCTC 49039=NBRC 113062)	23	[11]
*Chlorobaculum limnaeum* Imhoff 2003, 950^10^	sp. nov.	DSM 1677 (=NBRC 108881)^11^	28	[12]
*Coprococcus phoceensis* Bonnet *et al*. 2019, 3^3,12^	sp. nov.^13^	Marseille-P3062 (=DSM 103635=JCM 37525)^14^	41	[13]
*Corynebacterium neomassiliense* Boxberger *et al*. 2020, 4^3,15^	sp. nov.	Marseille-P3888 (=CCUG 72352=CSUR P3888)	5	[14]
*Curtobacterium aurantiacum* Osdaghi *et al*. 2024, 9^3,16^	sp. nov.	50R (=CFBP 8819=ICMP 22071)	39	[15]
*Endosaccharibacter* Pitiwittayakul *et al*. 2024, 9^3^	gen. nov.	*Endosaccharibacter trunci*	2	[16]
*Endosaccharibacter trunci* Pitiwittayakul *et al*. 2024, 10^3^	sp. nov.	KSS8 (=KCTC 92115=LMG 32414=NBRC 115232=TBRC 14669)	2	[16]
*Entomoplasma entomophilum* (Tully *et al*. 1988) Gasparich and Kuo 2019, 2737	comb. nov. [basonym: *Acholeplasma entomophilum* Tully *et al*. 1988]^17^	TAC (=ATCC 43706=NCTC 11713)	19	[17]
*Entomoplasma florum* (McCoy *et al*. 1984) Gasparich and Kuo 2019, 2737	comb. nov. [basonym: *Acholeplasma florum* McCoy *et al*. 1984]^17^	L1 (=ATCC 33453=NCTC 11704)	31	[17]
*Evansella alkalicola* (Zhai *et al*. 2022) Narsing Rao *et al*. 2022, 2^3^	comb. nov. [basonym: *Bacillus alkalicola* Zhai *et al*. 2022]	Zby6 (=JCM 17098=NBRC 107743)^18^	16	[18]
*Facivitalis* Arkan-Ozdemir *et al*. 2025, 10^3^	gen. nov.	*Facivitalis istanbulensis*	46	[19]
*Facivitalis istanbulensis* Arkan-Ozdemir *et al*. 2025, 10^3^	sp. nov.	JETA1-E2 (=DSM 117971=LMG 33634=KUEN 1206 (B) F3-1-1)	46	[19]
*Gaetbulibacter jejuensis* Oh *et al*. 2010, 309^19^	sp. nov.	CNURIC014 (=JCM 15976=KCTC 22615)	22	[20]
*Geochorda* Karnachuk *et al*. 2024, 10^3^	gen. nov.	*Geochorda subterranea*	33	[10]
*Geochorda subterranea* Karnachuk *et al*. 2024, 10^3^	sp. nov.	LN (=JCM 39508=VKM B-3703)	33	[10]
*Geochordaceae* Karnachuk *et al*. 2024, 10^3^	fam. nov.	*Geochorda*	33	[10]
*Haloarcula brevis* Ma *et al*. 2024, 10^3^	sp. nov.	DT43 (=CGMCC 1.18924=JCM 36146)	18	[21]
*Haloarcula regularis* Ma *et al*. 2024, 10^3^	sp. nov.	SYNS111 (=CGMCC 1.62601=JCM 36149)	18	[21]
*Haloarcula sediminis* Ma *et al*. 2024, 10^3^	sp. nov.	CK38 (=CGMCC 1.62732=JCM 36675)	18	[21]
*Halogeometricum luteum* Straková *et al*. 2024, 18^3^	sp. nov.	S3BR25-2 (=CCM 9253=CECT 30622)	4	[22]
*Halogeometricum salsisoli* Straková *et al*. 2024, 17^3^	sp. nov.	S1BR25-6 (=CCM 9250=CECT 30624)	4	[22]
*Haloplanus halobius* Zhang *et al*. 2024, 8^3^	sp. nov.	XH21 (=CGMCC 1.17029=JCM 34152)	13	[23]
*Haloplanus halophilus* Zhang *et al*. 2024, 8^3^	sp. nov.	GDY1 (=CGMCC 1.17472=JCM 34313)	13	[23]
*Haloplanus pelagicus* Zhang *et al*. 2024, 8^3^	sp. nov.	HW8-1 (=CGMCC 1.16850=JCM 34151)	13	[23]
*Haloplanus salilacus* Zhang *et al*. 2024, 7^3^	sp. nov.	CK5-1 (=CGMCC 1.17177=JCM 34153)	13	[23]
*Haloterrigena salinisoli* Dong *et al*. 2024, 11^3^	sp. nov.	KLK7 (=KCTC 4307=MCCC 4K00128)	9	[24]
*Halovalidus* Dong *et al*. 2024, 10^3^	gen. nov.	*Halovalidus salilacus*	9	[24]
*Halovalidus salilacus* Dong *et al*. 2024, 10^3^	sp. nov.	CCL63 (=CGMCC 1.18663=JCM 35096)	9	[24]
*Helicobacter cappadocius* Aydin *et al*. 2024, 11^3^	sp. nov.	faydin-H75 (=DSM 117062=LMG 33382=NCTC 14972)	7	[25]
*Hohaiivirga* Wang *et al*. 2024, 7^3^	gen. nov.	*Hohaiivirga grylli*	10	[26]
*Hohaiivirga grylli* Wang *et al*. 2024, 7^3^	sp. nov.	WL0021 (=GDMCC 1.2420=JCM 34655=MCCC 1K05886)	10	[26]
*Janthinobacterium kumbetense* Inan Bektas *et al*. 2023, 5^3,20^	sp. nov.	GK (=DSM 114233=LMG 32662)	12	[27]
*Levilactobacillus tujiorum* Gu 2024, 1^3^	sp. nov.	HBUAS51241 (=GDMCC 1.3022=JCM 35210)	38	[28]
*Limosilactobacillus allomucosae* Chen *et al*. 2024, 9^3^	sp. nov.	WILCCON 0055 (=DSM 117632=LMG 33563)	26	[29]
*Lutimaribacter degradans* Gao *et al*. 2023, 8^3^	sp. nov.	EGI FJ00013 (=CGMCC 1.19477=KCTC 92212)	6	[30]
*Mariniflexile litorale* Romanenko *et al*. 2024, 13^3^	sp. nov.	KMM 9835 (=KCTC 92792)	14	[31]
*Marinobacter sediminicola* An *et al*. 2024, 6^3^	sp. nov.	F26243 (=KCTC 92640=MCCC 1H01345)	43	[32]
*Marinobacter xiaoshiensis* An *et al*. 2024, 7^3^	sp. nov.	F60267 (=KCTC 92638=MCCC 1H01346)	43	[32]
*Methyloraptor* Saltykova *et al*. 2025, 11^3^	gen. nov.	*Methyloraptor flagellatus*	47	[33]
*Methyloraptor flagellatus* Saltykova *et al*. 2025, 11^3^	sp. nov.	S20 (=KCTC 8649=VKM B-3853)	47	[33]
*Microbacterium albipurpureum* corrig. Tsavkelova *et al*. 2024, 15^3,21^	sp. nov.	ET2 (=CGMCC 1.65005=VKM Ac-2998=VKPM Ac-2212)	36	[34]
*Natronobacterium haloterrestre* (Hezayen *et al*. 2002) Dong *et al*. 2024, 9^3^	comb. nov. [basonym: *Halobiforma haloterrestris* Hezayen *et al*. 2002]	135 (=DSM 13078=JCM 11627)	9	[24]
*Natronobacterium lacisalsi* (Xu *et al*. 2005) Dong *et al*. 2024, 10^3^	comb. nov. [basonym: *Halobiforma lacisalsi* Xu *et al*. 2005]	AJ5 (=CGMCC 1.3738=JCM 12983)	9	[24]
*Natronococcus wangiae* Dong *et al*. 2024, 10^3^	sp. nov.	AD-5 (=CGMCC 1.13783=JCM 33734)	9	[24]
*Natronococcus zhouii* Dong *et al*. 2024, 11^3^	sp. nov.	CG52 (=CGMCC 1.17139=JCM 34160)	9	[24]
*Parasalinivibrio* Er *et al*. 2025, 13^3^	gen. nov.	*Parasalinivibrio latis*	48	[35]
*Parasalinivibrio latis* Er *et al*. 2025, 13^3^	sp. nov.	TLL-SE01 (=BCRC 81435=JCM 36283)	48	[35]
*Parasutterella muris* Afrizal *et al*. 2022, e21^3^	sp. nov.	CLA-SR-150 (=DSM 111000=JCM 37186)	29	[5]
*Pedobacter jeongneungensis* Jung and Park 2012, 663	sp. nov.	BH45 (=JCM 17626=KACC 15514)	24	[36]
*Peribacillus aracenensis* Gutierrez-Albanchez *et al*. 2024, 5^3,23^	sp. nov.	BBB004 (=CCUG 76477=LMG 32742)^24^	42	[37]
*Plebeiibacterium* corrig. Yu *et al*. 2023, 10^3,25^	gen. nov.	*Plebeiibacterium marinum* corrig.	45	[38]
*Plebeiibacterium marinum* corrig. Yu *et al*. 2023, 10^3,26^	sp. nov.	D04 (=KCTC 92026=MCCC 1H00493)	45	[38]
*Plebeiibacterium sediminum* corrig. Yu *et al*. 2023, 12^3^	sp. nov.	AAT (=KCTC 92028=MCCC 1H00485)	45	[38]
*Prosthecodimorpha* Vasilyeva *et al*. 2024, 989	gen. nov.	*Prosthecodimorpha hirschii*	37	[39]
*Prosthecodimorpha hirschii* (Staley 1984) Vasilyeva *et al*. 2024, 989	comb. nov. [basonym: *Prosthecomicrobium hirschii* Staley 1984]	16 (=ATCC 27832=UQM 41824)^27^	37	[39]
*Prosthecodimorpha staleyi* Vasilyeva *et al*. 2024, 990	sp. nov.	22 (=DSM 113594=UQM 41461)^28^	37	[39]
*Pyxidicoccus parkwayensis* corrig. Ahearne *et al*. 2023, 13^3,29^	sp. nov.	SCPEA02 (=ATCC TSD-328=NRRL B-65670^30^	35	[40]
*Rhizomonospora* Yoon and Tamura 2024, 8^3^	gen. nov.	*Rhizomonospora bruguierae*	17	[41]
*Rhizomonospora bruguierae* Yoon and Tamura 2024, 8^3^	sp. nov.	TS60-4C (=NBRC 107566=TBRC 2024)	17	[41]
*Rhizosaccharibacter* Pitiwittayakul *et al*. 2024, 10^3^	gen. nov.	*Rhizosaccharibacter radicis*	2	[16]
*Rhizosaccharibacter radicis* Pitiwittayakul *et al*. 2024, 10^3^	sp. nov.	KSS12 (=KCTC 82433=LMG 32137=NBRC 114898=TBRC 13066)	2	[16]
*Rhodanobacter geophilus* Woo *et al*. 2024, 14^3^	sp. nov.	S2-g (=KACC 23733=TBRC 19012)	49	[42]
*Rhodanobacter lycopersici* Woo *et al*. 2024, 14^3^	sp. nov.	Si-c (=KACC 23732=TBRC 19125)	49	[42]
*Rhodococcus sacchari* Zang *et al*. 2024, 15^3^	sp. nov.	Z13 (=CCTCC AB 2022327=JCM 35797)	11	[43]
*Roseobacter sinensis* Wang *et al*. 2025, 8^3^	sp. nov.	WL0113 (=GDMCC 1.3082=JCM 33567)	40	[44]
*Saccharopolyspora galaxeae* Xie *et al*. 2024, 9^3^	sp. nov.	HNM0986 (=CCTCC AA 2020011=KCTC 49524)	32	[45]
*Saccharopolyspora montiporae* Xie *et al*. 2024, 7^3^	sp. nov.	HNM0983 (=CCTCC AA 2020014=KCTC 49526)	32	[45]
*Salinirarus* Zhang *et al*. 2024, 7^3^	gen. nov.	*Salinirarus marinus*	13	[23]
*Salinirarus marinus* Zhang *et al*. 2024, 7^3^	sp. nov.	XH8 (=CGMCC 1.17473=JCM 34314)	13	[23]
*Senimuribacter* Afrizal *et al*. 2022, e22	gen. nov.	*Senimuribacter intestinalis*	29	[5]
*Senimuribacter intestinalis* Afrizal *et al*. 2022, e22	sp. nov.	YCFAG-7-CC-SB-Schm-I (=DSM 106208=JCM 37188)	29	[5]
*Shumkonia* Yang *et al*. 2023, 1368	gen. nov.	*Shumkonia mesophila*	1	[46]
*Shumkonia mesophila* Yang *et al*. 2023, 1370^31^	sp. nov.	Y-P2 (=CCAM 826=JCM 34766)	1	[46]
*Shumkoniaceae* Yang *et al*. 2023, 1368	fam. nov.	*Shumkonia*	1	[46]
*Sorangium atrum* corrig. Ahearne *et al*. 2023, 13^3,32^	sp. nov.	WIWO2 (=NCCB 100922=NRRL B-65671)	35	[40]
*Sphingopyxis kveilinensis* Chen *et al*. 2024, 6^3^	sp. nov.	TUF1 (=CGMCC 1.62043=JCM 36394)	8	[47]
*Stigmatella ashevillensis* corrig. Ahearne *et al*. 2023, 14^3,33^	sp. nov.	NCWAL01 (=ATCC TSD-329=NCCB 100923)^34^	35	[40]
*Thiofractor* Makita *et al*. 2012, 792^35^	gen. nov.	*Thiofractor thiocamini* corrig.	25	[48]
*Thiofractor thiocamini* corrig. Makita *et al*. 2012, 792^36^	sp. nov.	496Chim (=DSM 22050=JCM 15747=NBRC 105224)	25	[48]
*Xenorhabdus bharatensis* Thanwisai *et al*. 2025, 8^3^	sp. nov.	CS20 (=CCM 9320=CCOS 2070)	44	[49]
*Xenorhabdus entomophaga* Thanwisai *et al*. 2025, 9^3^	sp. nov.	XENO-11 (=CCM 9389=CCOS 2111)	44	[49]
*Xenorhabdus siamensis* Thanwisai *et al*. 2025, 9^3,37^	sp. nov.	AUT15.5 (=CCM 9405=CCOS 2116)	44	[49]
*Xenorhabdus thailandensis* Thanwisai *et al*. 2025, 9^3,37^	sp. nov.	CCN3.3 (=CCM 9406=CCOS 2115)	44	[49]
*Xylanibacter caecicola* Afrizal *et al*. 2022, e25^3^	sp. nov.	PCHR (=DSM 105245=JCM 34700)	29	[5]

For references to Validation Lists 1–71, see *Int J Syst Bacteriol* **49** (1999) 1325. Lists 72–197 were published in *Int J Syst Evol Microbiol* **50** (2000) 3, 423, 949, 1415, 1699, 1953; and **51** (2001) 1, 263, 793, 1229, 1619, 1945; and **52** (2002) 3, 685, 1075, 1437, 1915; and **53** (2003) 1, 373, 627, 935, 1219, 1701; and **54** (2004) 1, 307, 631, 1005, 1425, 1909; and **55** (2005) 1, 547, 983, 1395, 1743, 2235; and **56** (2006) 1, 499, 925, 1459, 2025, 2507; and **57** (2007) 1, 433, 893, 1371, 1933, 2449; and **58** (2008) 1, 529, 1057, 1511, 1993, 2471; and **59** (2009) 1, 451, 923, 1555, 2129, 2647; and **60** (2010) 1, 469, 1009, 1477, 1985, 2509; and **61** (2011) 1, 475, 1011, 1499, 2025, 2563; and **62** (2012) 1, 473, 1017, 1443, 2045, 2549; and **63** (2013) 1, 797, 1577, 2365, 3131, 3931; and **64** (2014) 1, 693, 1455, 2184, 3603; and **65** (2015) 1, 741, 1112, 2017, 2777, 3767; and **66** (2016) 1, 1603, 1913, 2463, 3761, 4299; and **67** (2017) 1, 529, 1095, 2075, 3140, 4291; and **68** (2018) 1, 693, 1411, 2130, 2707, 3379; and **69** (2019) 5, 597, 1247, 1844, 2627, 3313; and **70** (2020) 1, 1443, 2960, 4043, 4844, 5596. Lists 197–221 were published in *Int J Syst Evol Microbiol* **71** (2021) 004600, 004688, 004773, 004846, 004943, 005096; and **72** (2022) 005167, 005260, 005331, 005422, 005517, 005592; and **73** (2023) 005709, 005812, 005845, 005931, 005997, 006080; and **74** (2024) 006173, 006229, 006275, 006398, 006452, 006501; and **75** (2025) 006562.

1 Abbreviations of culture collections cited in this list can be found at https://lpsn.dsmz.de/text/collections.

2 Priority number is assigned according to the date the documentation and request for validation are received.

3 The journal in which the name was effectively published does not have continuous pages.

4 Type and culture collection deposits were not given in the protologue. The type designation is found in the paper, and culture collection deposits are listed in the abstract.

5 The preferred etymology is Arabic masc. n. *al-qalyi*, the ashes of saltwort; N.L. neut. n. *alkali*, alkali; N.L. neut. dim. n. *spirillum*, a small spiral; N.L. neut. n. *Alkalispirillum*, an alkaline-loving spiral.

6 The authors gave *Alkalispirillum mobile* DSM 12769 as the type.

7 The etymology must state L. fem. adj. instead of L. masc. adj.

8 The list editors have corrected *Carboxydochorda* to *Carboxydichorda* (Car.bo.xy.chor’da. N.L. neut. n. *carboxydum* …).

9 The protologue heading must state *Cellulosimicrobium arenosum* sp. nov. instead of Cellulosimicrobium Arenosum sp. Nov.

10 The etymology must state N.L. neut. adj. instead of M.L. neut. adj.

11 The name was previously effectively but not validly published in the *IJSEM*.

12 The etymology must state L. masc. adj. instead of L. neut. adj.

13 Erroneously given in the article title as gen. nov., sp. nov.

14 The effective publication states that the type strain was also deposited as CSUR P3062, but no documentation was supplied.

15 The preferred etymology is ne.o.mas.si.li.en’se. Gr. masc. adj. *neos*, new; L. neut. adj. *massiliense*, pertaining to Massilia, the Roman name of Marseille, and the specific epithet of a *Corynebacterium* species; N.L. neut. adj. *neomassiliense*, a new (*Corynebacterium*) *massiliense.*

16 The protologue heading must state sp. nov. instead of sp. Nov.

17 The name was previously effectively but not validly published in the *IJSEM*.

18 The effective publication states that the type strain was also deposited as CGMCC 1.10368, but no documentation was supplied.

19 The etymology must state N.L. masc. adj. instead of N.L. fem. adj.

20 The protologue heading should state Description of *Janthinobacterium kumbetense* sp. nov. instead of Description of *J. kumbetense* sp. Nov.

21 The list editors have corrected *albopurpureum* to *albipurpureum* (al.bi.pur.pu’re.um … N.L. neut. n. *albipurpureum* …).

22 Deleted in proof.

23 The preferred syllabification is a.ra.cen.en’sis.

24 In the protologue, the CCUG number is given as CUGG76477.

25 The list editors have corrected *Plebeiobacterium* to *Plebeiibacterium* (Ple.bei.i.bac.te’ri.um. L. masc. adj. *plebeius*, plebeian, vulgar; N.L. neut. n. *bacterium*, a rod; N.L. neut. n. *Plebeiibacterium*, a vulgar rod-shaped bacterium).

26 The etymology must state L. neut. adj. *marinum*.

27 The effective publication states that the type strain was also deposited as DSM 8907, but no documentation was supplied.

28 The effective publication states that the type strain was also deposited as VKM B-3756, but no documentation was supplied.

29 The list editors have corrected *parkwaysis* to *parkwayensis* (park.way.en’sis. N.L. masc. adj. *parkwayensis*, pertaining to Parkway Farm in Landrum, SC, USA).

30 The authors gave TSD-328 instead of ATCC TSD-328.

31 The preferred syllabification is me.so’phi.la.

32 The list editors have corrected *aterium* to *atrum* (a’trum. L. neut. adj. *atrum*, black).

33 The list editors have corrected *ashevillena* to *ashevillensis* (a.she.vil.len’sis. N.L. fem. adj. *ashevillensis*, pertaining to Asheville, NC, USA).

34 The authors gave TSD-329 instead of ATCC TSD-329.

35 The preferred syllabification and etymology are Thi.o.frac’tor. Gr. neut. n. *theion*, sulfur; …

36 The list editors propose correcting the epithet to *thiocamini* (thi.o.ca.mi’ni. Gr. neut. n. *theion*, sulfur; L. masc. n. *caminus*, forge, melting furnace; N.L. gen. n. *thiocamini*, of a sulfur-rich chimney).

37 The preferred etymology is N.L. fem. adj. instead of N.L. masc./fem. adj.

## Abbreviations

ICNP: International Code of Nomenclature of Prokaryotes
*IJSEM*: *International Journal of Systematic and Evolutionary Microbiology*
